# Risks Associated with Dietary Exposure to Contaminants from Foods Obtained from Marine and Fresh Water, Including Aquaculture

**DOI:** 10.3390/ijerph23010085

**Published:** 2026-01-07

**Authors:** Martin Rose

**Affiliations:** Jorvik Food and Environmental Chemical Safety Ltd. (JFECS), York YO32 5YL, UK; martin.d.rose@outlook.com

**Keywords:** fish, seafood, risk assessment, residues and contaminants, aquaculture, radionuclides, micro- and nano-plastics, climate change

## Abstract

**Highlights:**

**Public health relevance—How does this work relate to a public health issue?**

- Fish and other foods produced in aquatic systems can be nutritious, but they are also prone to contain relatively high levels of chemical contaminants.

**Public health significance—Why is this work of significance to public health?**

- Increased aquaculture and recommendations to consume more fish may have an impact on dietary exposure to pollutants and contaminants found in fish and other products produced in these environments.

**Public health implications—What are the key implications or messages for practitioners, policy makers and/or researchers in public health?**

- Risk–benefit analysis should support recommendations to consume more fish.
- Regulatory control should be considered for emerging contaminants such as micro- and nano-plastics.

**Abstract:**

Aquatic environments have been a critical source of nutrition for millennia, with wild fisheries supplying protein and nutrients to populations worldwide. A notable shift has occurred in recent decades with the expansion of aquaculture, now representing a fast-growing sector in food production. Aquaculture plays a key role in mitigating the depletion of wild fish stocks and addressing issues related to overfishing. Despite its potential benefits, the sustainability of both wild and farmed aquatic food systems is challenged by anthropogenic pollution. Contaminants from agricultural runoff, industrial discharges, and domestic effluents enter freshwater systems and eventually reach marine environments, where they may be transported globally through ocean currents. Maintaining water quality is paramount to food safety, environmental integrity, and long-term food security. In addition to conventional seafood products such as fish and shellfish, foods such as those derived from microalgae are gaining attention in Western markets for their high nutritional value and potential functional properties. These organisms have been consumed in Asia for generations and are now being explored as sustainable foods and ingredients as an alternative source of protein. Contaminants in aquatic food products include residues of agrochemicals, persistent organic pollutants (POPs) such as dioxins, polychlorinated biphenyls (PCBs), and per- and polyfluoroalkyl substances (PFASs), as well as brominated flame retardants and heavy metals. Public and scientific attention has intensified around plastic pollution, particularly microplastics and nanoplastics, which are increasingly detected in aquatic organisms and are the subject of ongoing toxicological and ecological risk assessments. While the presence of these hazards necessitates robust risk assessment and regulatory oversight, it is important to balance these concerns against the health benefits of aquatic foods, which are rich in omega-3 fatty acids, high-quality proteins, vitamins, and trace elements. Furthermore, beyond direct human health implications, the environmental impact of pollutant sources must be addressed through integrated management approaches to ensure the long-term sustainability of aquatic ecosystems and the food systems they support. This review covers regulatory frameworks, risk assessments, and management issues relating to aquatic environments, including the impact of climate change. It aims to serve as a comprehensive resource for researchers, policymakers, food businesses who harvest food from aquatic systems and other stakeholders.

## 1. Introduction

The acceleration of global demand for aquatic-derived foods, alongside the proliferation of aquaculture activities and anthropogenic pressures, has led to a complex scenario of chemical contamination in aquatic environments [1,2]. The broad spectrum of pollutants and contaminants found in wild and farmed fishery products—including microplastics, nanoplastics, veterinary pharmaceuticals, pesticides, persistent organic pollutants (POPs), heavy metals, and compounds arising from eutrophication—demonstrates the intricate interplay of natural and human-driven processes [3,4]. Changing climatic conditions, such as rising sea temperatures and more frequent flooding events, further accelerate the spread and transformation of hazardous compounds in aquatic ecosystems, intensifying risks to food safety and public health [5,6].

This review provides an overview of key contaminants, their sources, environmental fate, and mechanisms of bioaccumulation and trophic transfer. The emerging risks associated with microplastics and nanoplastics are discussed with reference to their prevalence and poorly understood toxicity, which together present significant research challenges [7,8]. Notable knowledge gaps remain regarding plastic identification and behaviour in environmental mixtures, their interaction with adsorbed chemical pollutants, and the influence this exerts on bioavailability to aquatic organisms and humans [9]. Similarly, the growing recognition of algae as a resource for sustainable animal feeds and human foods requires comprehensive scientific scrutiny to guarantee the safety and functionality of derived products [10].

The impact of climate change is particularly significant for the biogeochemical cycling of mercury and the increased mobilisation of contaminants during flooding events [11,12]. Elevated sea surface temperatures catalyse the methylation of inorganic mercury, raising levels of more toxic methylmercury in food webs and heightening the risk of human exposure via consumption [13]. Floodwaters act as vectors for the redistribution of pollutants—including dioxins, heavy metals, and hydrocarbons—from contaminated to previously unaffected areas, as documented in case studies such as the flooding of the Elbe and Mulde rivers and the aftermath of Hurricane Katrina [14,15,16,17]. Increased contaminant concentrations in agricultural products, such as floodplain-produced milk, highlight the interconnectedness of environmental and food safety [18].

The management of aquatic resources is challenged by the transboundary nature of principal river systems, which cross multiple nations and regulatory regimes [19]. The movement of pollutants across borders, combined with disparate environmental policies, increases diplomatic complexity and the risk of international conflict regarding water purity and scarcity [20]. Large-scale infrastructure projects such as dams and irrigation can reduce supplies for downstream users, especially as populations grow and demand for clean water increases [21]. Robust international agreements and collaborative management frameworks are therefore essential to mitigate drought risk, ensure fair resource allocation, and prevent disputes [22].

By analysing the multifactorial nature of aquatic contamination and its effects on ecosystem integrity, food safety, and public health, this review provides a basis for understanding the importance of integrated risk assessment, adaptive policy development, and sustainable management of aquatic environments [23].

The purpose of this comprehensive review is to cover both food safety and health aspects, while also examining the sustainability challenges facing aquatic food production systems. It brings these topics together with an extensive coverage of a wide range of contaminants, from heavy metals and persistent organic pollutants to microplastics, coupled with discussion on the nutritional benefits of these foods, to offer a balanced and holistic perspective. By highlighting the necessity of integrating regulatory frameworks, risk assessments, and integrated management of aquatic environments, it is intended to serve as a resource for researchers, policymakers, and stakeholders in the aquaculture and fisheries industries with a broader coverage that other published reviews on the topic.

## 2. Water Systems

### 2.1. Freshwater (Rivers, Lakes Etc.) v. Marine Environments

A key distinction between inland and marine aquatic systems in relation to pollutant dynamics lies in the relative volume of water they contain. Inland waters such as rivers, lakes, and ponds generally possess significantly smaller volumes than marine environments, resulting in reduced dilution capacity. Consequently, contaminants introduced through agricultural runoff or industrial effluents, whether directly or indirectly, typically occur at higher concentrations in inland systems. Upon discharge into marine environments, these pollutants undergo substantial dilution, leading to markedly lower concentrations. However, this pattern is not universal. Certain inland waters in remote or undisturbed regions may exhibit minimal contamination, while some semi-enclosed or low-flushing marine systems, such as the Baltic Sea can accumulate elevated pollutant loads due to restricted water exchange and inputs from highly industrialized or densely populated catchments.

### 2.2. Aquaculture and Farmed Fish v. Wild Fish

Both wild-caught and farmed fish can accumulate or eliminate contaminants, nutrients, and other constituents present in their aquatic environment or feed. Certain compounds, such as veterinary drugs incorporated into commercial aquafeeds, are typically metabolised and excreted. In contrast, persistent organic pollutants (POPs), including dioxins, polychlorinated biphenyls (PCBs), brominated flame retardants, and per- and polyfluoroalkyl substances (PFASs) are resistant to degradation and may bioaccumulate in fish tissues [24]. Under specific conditions, the bioaccumulation of toxic contaminants in farmed fish, bivalves, crustaceans, and molluscs may reach levels of concern for human health.

The concentration of contaminants in fish and shellfish is influenced by multiple factors, including species, developmental stage, geographic origin, capture season, and tissue type. Variability in contaminant levels is observed both within and between species of farmed and wild fish [24,25,26].

Aquaculture is defined as the controlled rearing or cultivation of aquatic organisms using techniques that enhance production beyond the natural capacity of the environment [27]. Marine aquaculture typically involves the cultivation of species such as oysters, clams, mussels, shrimp, and salmon in cages or structures positioned on or suspended above the seafloor. Freshwater aquaculture is more commonly practiced in ponds or recirculating systems and produces species such as catfish, trout, tilapia, and bass [28].

Intensive aquaculture systems, in which high densities of fish are maintained in confined conditions, enable precise control of feeding regimes, growth rates, and disease management. Commercial feeds are often formulated for optimal nutrition and may include medicated additives to promote fish health. While such control enhances production efficiency and biosecurity, it also increases the potential for chemical residues in aquaculture products. Wild fish, in contrast, are less likely to contain residues of veterinary drugs or pesticides but are subject to environmental contamination beyond human control, such as exposure to industrial pollutants and variable dietary sources.

Aquaculture systems are generally categorized as extensive, semi-intensive, or intensive. In extensive systems, fish rely primarily on natural food sources in lagoons or brackish waters. Semi-intensive systems supplement the natural diet with formulated feeds, while intensive systems depend almost entirely on specialized feeds [29].

Several studies have reported higher concentrations of contaminants such as pesticides, polybrominated diphenyl ethers (PBDEs), and PCBs in farmed fish compared with their wild counterparts, including species such as salmon, catfish, turbot, and sea bass [30]. However, standardization of sampling and analytical methodologies is required before robust comparisons between wild and farmed fish can be made [25].

Global aquaculture production reached over 90 million tonnes in 2012, including 67 million tonnes of fish and seafood intended for human consumption. Approximately 90% of global aquaculture output originates from Asia, with China alone accounting for nearly two-thirds of production. In contrast, Europe contributed less than 5% of global aquaculture production in the same year [31].

Although aquaculture provides an increasingly important source of protein and contributes significantly to global food security and national economies, it also poses environmental and food safety challenges. Effluent discharges containing nutrients, organic matter, and chemical residues (e.g., antibiotics and antiparasitic agents) can contaminate surrounding waters, potentially leading to bioaccumulation of pollutants in cultured organisms. Contaminants may also originate from the feed ingredients themselves, which can contain residues of veterinary drugs, POPs, pesticides, and heavy metals such as mercury, lead, cadmium, chromium, arsenic, and selenium [32].

Thus, both feed composition and water quality are critical control points influencing the chemical safety of aquaculture products. Sustainable aquaculture practices therefore require integrated monitoring of environmental conditions, feed inputs, and residue levels to ensure product safety and minimize ecological impact.

## 3. Pollutants and Contaminants

The classes of pollutants and contaminants included in this review were chosen to represent (i) a broad coverage of regulated and near regulated (those under consideration for regulation or with government advisories etc); (ii) those included on the Stockholm list of POPs; (iii) a range of classes to represent behaviour of different chemical pollutants in aquatic environments.

## 4. Pesticides and Veterinary Medicines

### 4.1. Pesticides

Pesticide residues in foods sourced from both marine and freshwater environments, including those produced through aquaculture, have emerged as a matter of increasing concern for food safety, public health, and environmental integrity. The widespread use of pesticides in terrestrial agriculture means that these chemicals often find their way into aquatic systems, either through direct application in aquaculture or, more commonly, via indirect pathways such as surface runoff, leaching, and atmospheric deposition. Once introduced into rivers, lakes, and coastal waters, pesticides can persist, accumulate, and ultimately enter the food web, affecting a wide range of organisms from plankton to top predators, including humans who consume aquatic-derived foods [7,33].

In freshwater environments, agricultural runoff is a primary conduit for pesticide contamination. During rainfall events, excess pesticides applied to crops are washed from fields into adjacent water bodies. These compounds may include organophosphates, carbamates, pyrethroids, and persistent organic pollutants (POPs) such as DDT, lindane, and chlordane. The persistence and toxicity of these chemicals are influenced by factors such as water temperature, pH, and organic matter content, which can vary widely between freshwater and marine settings. Once in the aquatic environment, pesticides may adsorb to sediments, dissolve in water, or bind to organic material, affecting their bioavailability and potential for uptake by aquatic organisms [12,33].

In marine ecosystems, the transport of pesticides from land to sea is facilitated by riverine discharge and estuarine mixing. Coastal areas, especially those adjacent to intensive agricultural zones, are particularly vulnerable to contamination. Pesticides introduced into the marine environment can impact not only wild fish populations but also shellfish, crustaceans, and other seafood species harvested for human consumption. Certain pesticides, such as organochlorines, are highly persistent and lipophilic, leading to bioaccumulation in fatty tissues and biomagnification through the food chain. This is of particular concern for apex predators and humans, who may be exposed to higher concentrations of residues via dietary intake [33].

Aquaculture systems are also at risk of pesticide contamination, even when pesticides are not directly applied. Aquaculture ponds, cages, and pens may be situated in environments receiving agricultural runoff, thereby exposing farmed species to a range of agrochemicals. Additionally, some pesticides are intentionally used within aquaculture operations as therapeutic agents to control parasites and disease outbreaks, such as the employment of organophosphates and pyrethroids for sea lice management in salmon farming. In these cases, the compounds are reclassified as veterinary medicinal products, but their residue profiles and potential impacts on food safety remain significant. The use of such chemicals must be carefully regulated and monitored, as improper application can result in excessive residue accumulation in edible tissues, posing risks to consumers and leading to trade restrictions or economic losses [34,35,36].

Residues of pesticides in aquatic foods are typically monitored through national and international food safety programmes, with established maximum residue limits (MRLs) for various compounds. However, the diversity of pesticides, environmental conditions, and species involved complicates the assessment and management of risks. Chronic exposure to low levels of pesticide residues has been associated with a range of adverse health outcomes, including endocrine disruption, neurotoxicity, carcinogenicity, and developmental effects. Vulnerable populations, such as children, pregnant women, and communities with high seafood consumption, may be at heightened risk [7].

Mitigating pesticide residues in foods from aquatic environments requires a multifaceted approach. This includes reducing pesticide use in agriculture through integrated pest management (IPM), promoting buffer zones and riparian vegetation to limit runoff, and strengthening regulatory oversight of pesticide application in aquaculture. Advances in analytical techniques have improved the detection and quantification of pesticide residues, but continued research is needed to better understand the fate, transport, and transformation of these chemicals in complex aquatic systems. Moreover, public awareness and education are essential to promote responsible consumption and support policy development aimed at safeguarding food safety and environmental health [33,35].

### 4.2. Veterinary Medicines

Veterinary medicines play a critical role in both terrestrial and aquatic animal production systems, but their use also raises complex challenges for environmental and food safety. Pharmaceuticals administered to livestock are often excreted and end up deposited on agricultural land, from where they can be transported into freshwater environments. Human pharmaceuticals also contribute, entering aquatic systems via discharges from wastewater treatment plants, which are frequently unable to remove these compounds entirely [35].

In aquaculture, certain chemicals initially classified as pesticides, such as organophosphates and pyrethroids, are deliberately used as therapeutic agents to control ectoparasites like sea lice in salmonids. When used in this context, these compounds are reclassified as veterinary medicinal products (VMPs) [35,36].

Veterinary medicines in both terrestrial and aquatic systems are used for a range of purposes: disease prevention (e.g., vaccines), therapeutic interventions (e.g., antimicrobials and antiparasitics), and husbandry practices (e.g., anaesthetics, reproductive hormones, and disinfectants). While terrestrial farming typically focuses on a few species, aquaculture is highly diverse, involving over 500 cultured organisms including finfish, crustaceans, molluscs, amphibians, invertebrates, and macroalgae. This diversity complicates pharmaceutical development, as efficacy and safety data are often species-specific and insufficient, particularly for species with limited commercial value, which further restricts investment in drug development and regulatory approval [35].

As in terrestrial farming, VMPs in aquaculture are mainly used for the prophylaxis and treatment of infectious diseases. Antimicrobials are generally administered via medicated feed or bath immersion. Disease outbreaks often include both visibly ill and subclinical carriers, so metaphylactic treatment—where the entire population is medicated to treat existing infections and prevent further spread—is common [35].

Unlike terrestrial livestock production, where antimicrobials have historically been used at low doses to promote growth by altering gut microbiota, there is little evidence to support such effects in aquatic species. Growth promotion via antimicrobials is not a standard practice in aquaculture [37].

The rapid global expansion of aquaculture over the past thirty years has been supported by the use of veterinary medicines. However, the antimicrobials used in aquaculture are generally the same as those in human medicine, with no antibiotics developed exclusively for aquatic use. High drug development costs and limited market opportunities hinder innovation in this area [35].

With rising awareness of biosecurity and health management, the use of veterinary medicines in aquaculture has increased, but stewardship has not always been optimal. Residues of veterinary drugs have, at times, led to trade restrictions and economic losses when import regulations are breached [38]. The detection of chloramphenicol residues in shrimp since 2001 has triggered stricter regulation and oversight of antimicrobial use [36].

While essential for disease control, veterinary medicines have limitations. The development of vaccines has reduced reliance on antimicrobials in some aquaculture sectors. Nevertheless, misuse of VMPs can result in toxicological effects, antimicrobial resistance, environmental contamination, residue accumulation, and potential risks to public health when foods produced in such environments are consumed. Environmental factors such as water temperature, salinity, and organic load can influence drug effectiveness and persistence, complicating treatment and risk assessments [35].

Maintaining the health of aquatic animals in intensive culture systems requires access to effective VMPs to optimise survival, reduce disease-linked losses, and improve feed efficiency. There is broad agreement that veterinary medicines contribute significantly to aquaculture productivity, but a transition towards judicious, evidence-based use is vital. Strengthening regulations, improving farmer education and extension services, and promoting best practices in antimicrobial stewardship are key steps for sustainable aquaculture development [35].

The application of veterinary medicines in aquaculture, much like in terrestrial livestock and poultry systems, underpins the efficiency and sustainability of industrial-scale aquatic food production. Their strategic use boosts production efficiency by maximising the use of land, water, and feed resources per unit biomass, thereby supporting food security and economic viability [35]. The main ways in which veterinary medicines are used in modern aquaculture are summarised in Table 1.

Overall, veterinary medicines are a cornerstone of sustainable, resilient, and welfare-conscious aquaculture, enabling stable production and ensuring the sector’s long-term viability [34,35]. Problems associated with misuse and overuse of veterinary medicines are summarised in Table 2.

## 5. Persistent Organic Pollutants (POPs)

Persistent Organic Pollutants (POPs) are a class of organic compounds characterised by their resistance to environmental degradation through chemical, biological, and photolytic processes. Due to their inherent physicochemical stability, these substances persist in the environment for extended periods following their release, often spanning decades. POPs exhibit long-range environmental transport via atmospheric, aquatic, and terrestrial pathways, leading to global dispersion and deposition far from their original sources [39,40].

Most POPs are lipophilic and exhibit low water solubility, which facilitates their partitioning into biological lipid compartments. Consequently, they bioaccumulate in the adipose tissues of organisms and biomagnify through trophic levels, resulting in exponentially elevated concentrations (up to 70,000 times background levels) in top level predators such as some species of fish, birds of prey, marine mammals, and humans. POPs have been detected in biota and humans even in remote regions such as the Arctic, underscoring their capacity for long-range atmospheric and oceanic transport [40,41].

Chronic exposures to POPs have been linked to a spectrum of toxicological outcomes, including carcinogenicity, immunotoxicity, neurotoxicity, reproductive and developmental toxicity, and hypersensitivity reactions. Several POPs act as endocrine-disrupting chemicals (EDCs), perturbing hormonal homeostasis and thereby inducing adverse effects on reproductive capacity, immune function, and offspring development [42,43].

### 5.1. Origins and Global Impact

Environmental contamination by POPs has primarily resulted from industrial production, agricultural applications, and combustion processes over the decades following industrialisation. The persistence and bioaccumulative nature of these substances have led to sustained exposure across generations through contaminated food chains, particularly via consumption of fish and other lipid-rich foodstuffs [39,40].

### 5.2. The Stockholm Convention

The Stockholm Convention on Persistent Organic Pollutants is an international environmental treaty established to protect human health and the environment from POPs [39]. Adopted in 2001 and entering into force in 2004, the Convention initially targeted twelve chemicals—commonly referred to as the “Dirty Dozen”—classified into three functional categories: Pesticides: aldrin, chlordane, DDT, dieldrin, endrin, heptachlor, hexachlorobenzene, mirex, and toxaphene; Industrial chemicals: hexachlorobenzene and polychlorinated biphenyls (PCBs); Unintentional by-products: hexachlorobenzene, polychlorinated dibenzo-p-dioxins (PCDDs), polychlorinated dibenzofurans (PCDFs), and PCBs. Regulations for limits in foods including fish for many of these original pollutants have come into force in some parts of the world, e.g., for the pesticides and PCBs in many parts of the world, and for PCDD/Fs in the EU and some other places.

In 2009, the Convention expanded to include an additional 17 substances across the same three categories. Among these were brominated flame retardants (BFRs), such as polybrominated diphenyl ethers (PBDEs), and perfluorooctane sulfonic acid (PFOS), along with its salts and perfluorooctane sulfonyl fluoride (PFOSF), which belong to the broader group of per- and polyfluoroalkyl substances (PFASs), often referred to as “forever chemicals.” [39,44,45]. Regulations for these contaminants are not yet as well established, but are starting to come into force, e.g., limits for PFASs in food in the EU.

PFOS is both an intentionally produced compound and an unintentional degradation product of other fluorinated substances. It has been widely used in electronic components, fire-fighting foams, photographic processes, hydraulic fluids, and textiles. Although restrictions on PFOS production and use have increased globally, it continues to be manufactured in several countries. Industry has increasingly shifted toward shorter-chain PFAS analogues, which, despite reduced bioaccumulative potential, retain significant persistence, mobility, and toxicity [44,45].

Unlike traditional POPs, many PFASs do not preferentially partition into lipids but instead bind strongly to serum proteins and hepatic tissues. This alternative mechanism of bioaccumulation nonetheless leads to sustained internal exposure and biomagnification through food webs. Given their extreme environmental persistence, potential for long-range transport, and demonstrated toxicity, certain PFASs meet the Annex D criteria for inclusion under the Stockholm Convention [39,44].

Concentrations of POPs in foods including fish and other products produced in aquatic systems that give rise to health concerns vary considerably. Parts per million or parts per billion (mg/kg–μg/kg range) may be important for some of the pesticides a = but concentrations in the low parts per trillion (ng/kg–pg/kg range) are important for other POPs such as PCDD/Fs and PFASs.

## 6. POPs in Fish and Seafood

The principal source of persistent organic pollutants (POPs) in aquaculture-derived fish and seafood products is contaminated feed, whereas in wild-caught seafood, primary inputs originate directly from anthropogenic activities, including industrial emissions, improper disposal of POP-containing materials, and the use of POP-contaminated by-products. These compounds are introduced into aquatic environments through multiple pathways, such as surface runoff from agricultural and industrial lands, atmospheric deposition, effluent discharge from industrial processes, and leaching from landfills [40].

To assess the environmental behaviour and biological impacts of POPs, extensive monitoring and analytical studies have been conducted across trophic levels ranging from invertebrates to higher vertebrates, in diverse geographic regions including the Arctic, North America, Asia, and Europe. Over several decades, these investigations have elucidated the bioaccumulation dynamics, sources, metabolic transformations, and toxicological effects of POPs in aquatic organisms [41].

Trophic transfer processes, including bioaccumulation and biomagnification, result in elevated POP concentrations in apex (top-level) predators. Numerous studies have documented a spectrum of adverse effects associated with these contaminants, such as endocrine disruption, developmental and reproductive toxicity, and immunotoxic responses, particularly in species occupying the highest trophic positions (e.g., marine mammals, predatory fish, and lipid-rich pelagic fish) [42,46].

As measures have been introduced for established POPs, e.g., pollution control measures to address production and environmental release of PCDD/Fs and restrictions on production and use of brominated flame retardants such as PBDEs, concentrations in the environment and subsequently food including fish have been falling. However, as a result of regrettable substitution (see later) new POPs are being introduced into the market and concentrations of these emerging pollutants are increasing.

Furthermore, climate change has been identified as a compounding stressor for contaminant-exposed populations, influencing POP distribution, environmental persistence, and toxicity through alterations in food web structure, temperature regimes, and biogeochemical cycling [47].

Consequently, fish and seafood consumption represents a major pathway for human dietary exposure to POPs in many populations worldwide [40,41,42].

## 7. Metal(oid)s in Fish

Metal(loid)s are widespread in aquatic environments as a result of both natural geochemical processes and human activities such as industrial discharge, mining, and agricultural runoff [48]. Fish can accumulate these elements either directly from water and sediments or indirectly through their diet, in both wild and aquaculture settings [49]. The primary route for dissolved metal ions is absorption through the gill epithelium and other permeable tissues, while particle-bound metals are ingested, dissociated within the digestive tract, and then absorbed via the intestinal epithelium [50,51]. The distribution of metals within fish tissues is not uniform; typically, the liver accumulates higher concentrations than muscle tissue [52]. Several trace elements, such as iron, zinc, copper, cobalt, manganese, chromium, selenium, and nickel, are essential micronutrients for normal physiological and metabolic processes in fish, but excessive accumulation can lead to toxicity.

**Figure 1:**
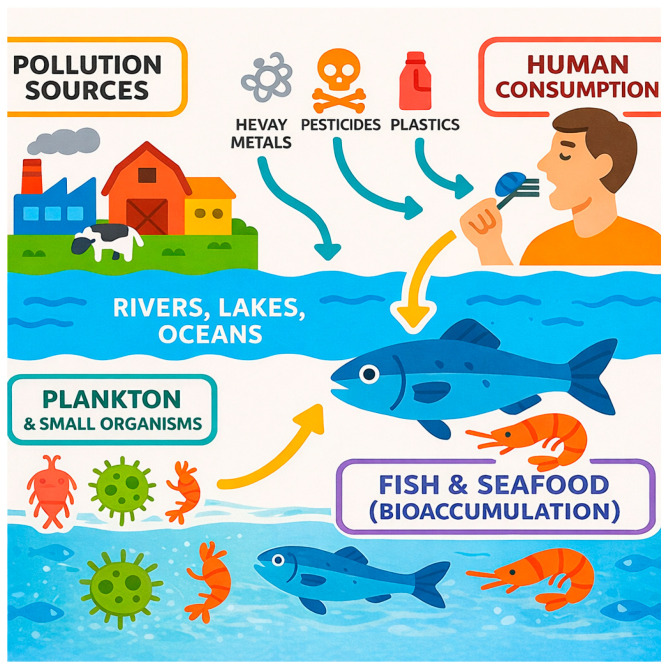
Contamination pathways for fish and seafood.

Contamination levels vary among fish species, reflecting differences in habitat, feeding behavior, and position in the food web. For example, rainbow trout (*Oncorhynchus mykiss*) [53] exposed to high levels of metals may develop deformities, while European sea bass (*Dicentrarchus labrax*) [54] can experience growth inhibition under similar conditions. In common carp (*Cyprinus carpio*), elevated metal concentrations have been linked to reduced hatching success and deformities in larvae [55]. Marine organisms, in general, tend to accumulate higher levels of arsenic, particularly in organic forms, compared to freshwater species. This is due to their unique ability to biotransform inorganic arsenic into organoarsenic compounds. Over 70 distinct organic arsenic species have been identified in marine systems [56,57]. Predatory fish, such as tuna and swordfish, often exhibit higher concentrations of mercury, especially methylmercury, due to biomagnification through the aquatic food web. Simplistic representations of contamination pathways for fish and seafood are shown in Figure 1.

The health effects of metal(loid) exposure are diverse and depend on the specific element involved. In fish, toxicity can manifest as increased mortality, developmental deformities, growth inhibition, and impaired reproduction. Certain metals have organ-specific effects: lead, cadmium, and mercury can impair renal function; lead and cadmium may cause liver damage; and mercury and lead are associated with neurological deficits [58,59]. In humans, the consumption of contaminated fish and seafood poses significant risks, particularly from mercury (notably methylmercury) and arsenic [60]. Methylmercury is especially concerning due to its persistence, and tendency to bioaccumulate and biomagnify [61]. Human exposure to methylmercury occurs mainly through the consumption of fish and shellfish, and can result in neurological symptoms such as paresthesia, ataxia, tremors, and impaired coordination [62]. While marine organisms often contain high levels of arsenic, most of it is present in organic forms like arsenobetaine, which are less toxic than inorganic arsenic [63].

Cooking and processing methods can influence the bioavailability of metals in fish consumed by humans. Boiling may reduce the content of some water-soluble metals, but it has limited effect on methylmercury and organoarsenic compounds, which are stable and remain in the cooked tissue. Frying and grilling generally do not significantly reduce mercury or arsenic levels. Removing the skin and fatty tissues can lower exposure to lipophilic contaminants such as methylmercury. Processing methods like canning and smoking may alter the chemical form of metals but often do not substantially decrease the total metal content. Therefore, while certain cooking practices can modestly reduce exposure to some metals, the most toxic forms—methylmercury and organoarsenic compounds—are largely unaffected. As a result, the most effective way to reduce health risks is to select fish species known to have lower contamination levels and to limit the consumption of high-risk species.

## 8. Radionuclides

Radioactive elements, both naturally occurring and anthropogenic, are present in the food supply. These radionuclides typically enter the food chain through widespread environmental contamination [64]. As with stable isotopes, radioactive species may be deposited onto terrestrial and aquatic environments via atmospheric fallout or from contaminated water sources. This leads to direct exposure of livestock and aquatic organisms, or alternatively, results in the uptake of radionuclides by plants from contaminated soils or surface deposition [65]. Consumption of such contaminated vegetation by farm animals subsequently leads to the presence of radionuclides in animal-derived food products, including meat.

### 8.1. Health Risks from Dietary Exposure to Radionuclides

Dietary exposure to radionuclides poses a range of potential health risks, primarily dependent on the type, dose, and duration of exposure as well as the chemical and radiological properties of the specific isotopes involved [64]. Ingested radionuclides can accumulate in various tissues and organs, where they emit ionising radiation that may damage cellular structures and DNA, thereby increasing the risk of cancer and other health disorders [66]. For example, caesium-137 is distributed throughout soft tissues, while strontium-90 tends to concentrate in bones, potentially elevating the risk of leukaemia and bone cancers [67]. Iodine-131, on the other hand, accumulates in the thyroid gland and has been linked to an increased incidence of thyroid cancer, particularly in children [64]. Chronic low-level exposure may also contribute to broader systemic effects, such as impaired immune function and increased rates of genetic mutations [65]. Although routine monitoring has thus far found limited evidence of adverse health outcomes from biomagnification of radionuclides in aquatic food webs, accidental releases and localised contamination events can result in significant, acute health hazards for exposed populations [66].

After ingestion, radionuclides may be absorbed in the digestive tract and distributed throughout the body, potentially increasing the risk of cancer and other health effects if exposure is prolonged or levels are high. Inhalation risks generally arise from airborne radionuclides released from industrial sources or nuclear accidents and are a smaller source of exposure for most of the population.

Regulatory agencies, such as the Food Standards Agency (FSA) in the UK and the European Food Safety Authority (EFSA), set limits for radionuclide concentrations in food products, including fish and shellfish. These standards are based on scientific risk assessments and are designed to protect public health. Commonly monitored radionuclides include caesium-137, strontium-90, and iodine-131.

Routine monitoring involves sampling fish and shellfish from various locations and analysing them using sensitive laboratory techniques, such as gamma spectrometry. Results are compared to regulatory limits, and actions are taken if levels exceed thresholds. Monitoring programmes also track trends over time to identify potential changes in environmental contamination and to inform risk management decisions.

International guidelines, such as those from the Codex Alimentarius, provide harmonised reference values to support trade and public health. These protocols ensure consistency and transparency in how radionuclide contamination is assessed and managed globally.

Effective risk communication is essential for maintaining public trust and enabling informed choices. Strategies include issuing clear public advisories when elevated radionuclide levels are detected, providing regular updates on monitoring results, and offering practical guidance on safe seafood consumption. Educational materials should explain what radionuclides are, how they can affect health, and what steps consumers can take to minimise exposure

### 8.2. Natural Radioisotopes

There are three types of natural radioactive elements in food: long-lived elements like uranium-238, thorium-232, and potassium-40; their daughter products such as radium-226, which can decay further into substances like radon, lead-210, and polonium-210; and isotopes created by cosmic rays, like carbon-14 from nitrogen [65]. Natural radioisotopes make up about a quarter of our background radiation exposure [66].

### 8.3. Artificial Radioisotopes

The prevalence of artificial radioisotopes in foodstuffs has markedly increased since the detonation of the Hiroshima atomic bomb in 1945, with further pronounced rises during the 1960s due to extensive atmospheric nuclear testing [65]. Additional sources include emissions from nuclear power stations, waste processing facilities, and medical and research activities [66]. Atomic detonations and operational reactors generate a complex mixture of radioactive elements, including uranium-235 and plutonium-239, as well as fission products like strontium-90, cobalt-60, ruthenium-106, caesium-137, and iodine-131 [64]. Activation products such as zinc-65 and carbon-14 are formed through neutron interactions with naturally present elements. Neutron bombardment in reactors also facilitates the production of radioisotopes for medicine and industry [66].

Differential uptake of radioactive isotopes is critical in assessing contamination. Plutonium compounds, though hazardous, have low plant uptake due to poor solubility, unlike caesium or strontium [65]. However, surface contamination and ingestion remain concerns. Aquatic discharges can lead to bioaccumulation in marine organisms—flatfish may concentrate caesium-137 by a factor of 20, and zinc-65 has been found in oysters near nuclear effluent outlets [64]. Surveillance programmes focus on fish and marine products, though no biomagnification-related health effects have been confirmed to date [66].

## 9. Incidents Involving Food Contamination by Radionuclides

### 9.1. Chernobyl

The Chernobyl accident in April 1986 released a substantial radioactive plume due to reactor containment failure. Over 30 immediate fatalities occurred, and 135,000 people were evacuated [65]. Elevated caesium-137 levels were found in fish and sheep in the UK and Norway. Although levels declined significantly in the first five years, reductions slowed thereafter. By 2011, UK movement controls on sheep were lifted due to negligible consumer risk, though restrictions on berries, mushrooms, and fish from parts of the former USSR remain [64].

### 9.2. Fukushima

On 11 March 2011, a magnitude 9.0 earthquake and tsunami caused reactor meltdowns at Fukushima, releasing radionuclides into the environment [67]. Regulatory limits were set to cap effective doses at 5 mSv/year and thyroid doses at 50 mSv/year. Contamination was found in water, milk, vegetables, and fish. Although beef generally remained below thresholds, livestock grazing and feed use were restricted. Trace radionuclides appeared globally, but contamination was mostly localised. Japan implemented extensive monitoring, and some import restrictions remain [68].

## 10. Natural Toxicants and Contaminants

Natural toxicants are also present in fish and other foods sourced from aquatic environments. These include biotoxins produced by certain algae, such as saxitoxins, domoic acid, and ciguatoxins, which can accumulate in shellfish and finfish through the food web. Consumption of seafood contaminated with these substances can lead to a range of illnesses, including paralytic, amnesic, and ciguatera fish poisoning, characterised by neurological and gastrointestinal symptoms [69,70]. Additionally, some fish species naturally contain high levels of histamine or tetrodotoxin, the latter being particularly associated with pufferfish and capable of causing severe or even fatal poisoning if ingested [71,72]. The occurrence of these toxicants is influenced by environmental factors such as water temperature, nutrient availability, and the presence of specific algal blooms, making ongoing monitoring essential to safeguard public health [73].

Concerns regarding mycotoxins in fish and seafood have been growing, particularly as aquaculture expands and the use of plant-derived feed ingredients increases. Mycotoxins, toxic secondary metabolites produced by certain fungi (mainly Aspergillus, Fusarium, and Penicillium species), can contaminate feedstuffs such as cereals, oilseeds, and other plant-based materials commonly incorporated into aquafeeds [32,74]. When fish and shellfish consume contaminated feed, mycotoxins may accumulate in their tissues, potentially posing risks to both aquatic animal health and human consumers [75].

Exposure to mycotoxins in fish can lead to impaired growth, weakened immune response, and increased susceptibility to disease, thereby impacting aquaculture productivity and sustainability [74,76]. For humans, the main concern is the potential transfer of these toxins through the food chain, as some mycotoxins are stable under typical cooking and processing conditions [77]. Chronic intake of contaminated seafood could contribute to adverse health effects, including immunosuppression, carcinogenicity, and organ toxicity [72,75]. As such, continuous monitoring of mycotoxin levels in aquafeeds and seafood products, alongside good storage and handling practices, is essential to minimise risks and safeguard public health [32,76].

## 11. Eutrophication

Eutrophication is a primary driver of harmful algal bloom formation, which relates directly to the previous section. The term refers to the enrichment of surface waters with nutrients, primarily those that stimulate plant and algal growth. Although this process can occur naturally, it is most commonly associated with anthropogenic nutrient inputs, particularly from agricultural activities [78]. The ‘trophic status’ of a lake, defined as the relationship between its nutrient concentration and the resulting production of organic matter, is a key parameter in lake management and ecological assessment. Eutrophication represents a transition from a lower to a higher trophic state, driven by increased nutrient availability [79].

Agricultural practices are a major contributor to eutrophication, as both nitrogen (N) and phosphorus (P) from fertilizers can be transported into surface waters through runoff and leaching. While both nutrients play a role, the classification of trophic status generally focuses on the limiting nutrient, typically phosphorus [78]. The influx of agricultural fertilizers into aquatic systems promotes excessive algal growth, leading to oxygen depletion and the formation of hypoxic or anoxic “dead zones” in downstream environments. In severe cases, such conditions can render aquatic habitats unsuitable for fish and other marine organisms [79,80].

Eutrophication also facilitates the proliferation of harmful algal bloom (HAB) species, many of which produce potent biotoxins that pose risks to human and animal health. These toxins can accumulate in marine food webs, particularly in shellfish, leading to conditions such as paralytic, neurotoxic, and diarrhoeic shellfish poisoning. Finfish may also serve as vectors, as observed in ciguatera fish poisoning, where predatory species bioaccumulate toxins initially produced by dinoflagellates [78]. Consumption of contaminated seafood by humans can result in a range of gastrointestinal and neurological symptoms, underscoring the broader ecological and public health implications of eutrophication [79].

## 12. Microplastics and Nanoplastics

It is estimated that approximately 70–80% of plastics entering the marine environment are transported via riverine systems, which act as major conduits between terrestrial sources and the ocean [81]. The primary contributors to this flux include industrial and agricultural activities, as well as effluent discharged from wastewater treatment plants [82,83,84].

Synthetic microfibres and microplastics are also generated during domestic laundering processes; for instance, a standard 5 kg wash load of polyester textiles can release up to 6 × 10^6^ microfibres [83]. Although over 95% of these fibres are typically retained within sewage sludge (biosolids), the subsequent application of these biosolids to agricultural land facilitates the re-emission of microplastics into the environment [82]. Through aeolian dispersion or surface water runoff, these particles can re-enter fluvial systems and eventually reach marine environments [84].

In contrast, marine-derived plastics originate from sources such as shipping, offshore oil and gas platforms, fishing activities, and recreational maritime pursuits (e.g., discarded fishing nets) [85]. Additionally, a substantial proportion of marine microplastics is derived from tyre wear particles, which are transported from roads into aquatic systems through stormwater drainage [85]. Many microplastic particles exhibit sizes and optical properties similar to planktonic organisms, increasing their likelihood of ingestion by marine biota [81].

A summary of the key contaminant classes found in fish and seafood is presented in Table 3, along with notable health risks for each class.

### 12.1. Bioaccumulation and Trophic Transfer

The ingestion of microplastics by fish and shellfish represents a key route of entry into the human food chain, as these organisms serve as important dietary protein sources globally [86]. Comparative studies have indicated that pelagic fish species, such as North Sea fish and Atlantic cod (*Gadus morhua*) from Newfoundland, exhibit relatively low microplastic burdens, whereas estuarine and freshwater species show higher levels of contamination [87]. For instance, a study of fish from a South American river found widespread presence of plastic debris in the gastrointestinal tract [87]. In the River Thames (London, UK), microplastics were detected in 75% of European flounder (*Platichthys flesus*), a demersal species, compared with 20% of European smelt (*Osmerus eperlanus*), a piscivorous species [86]. Similarly, zooplanktivorous species such as anchovy, pilchard, and herring have demonstrated the presence of microplastic particles in hepatic tissues [88]. Although microplastics tend to accumulate in the gills, liver, and gastrointestinal tract—tissues not generally consumed by humans—their presence in edible shellfish presents a more direct exposure route [86].

### 12.2. Uptake in Filter Feeders and Human Exposure

Filter-feeding organisms appear particularly susceptible to microplastic uptake due to the comparable size of microplastics and their natural food particles. Laboratory studies have shown that blue mussels (*Mytilus edulis*) exposed to nanoplastics (30–100 nm) exhibit significant intestinal absorption [89]. Furthermore, the trophic transfer of nanoplastics has been demonstrated from phytoplankton to zooplankton and subsequently to fish [90]. Analysis of soft tissues from commercially cultivated blue mussels and Pacific oysters (*Crassostrea gigas*) revealed microplastic concentrations of 36 ± 7 and 47 ± 16 particles per 100 g wet weight, respectively [91]. Since these organisms are typically consumed whole, it has been estimated that consumers of European shellfish may ingest approximately 11,000 microplastic particles annually, although this estimate likely overrepresents exposure for individuals who consume shellfish infrequently [91]. Experimental evidence suggests that 10 μm represents the upper size threshold for microplastic translocation into the circulatory system of mussels, providing an indicative size range that is likely to be relevant for potential human absorption [89].

### 12.3. Adsorption of Pollutants and Ecotoxicological Implications

Microplastic particles possess a high surface area-to-volume ratio, facilitating the adsorption of hydrophobic contaminants such as persistent organic pollutants (POPs), including dioxins, polychlorinated biphenyls (PCBs), and brominated flame retardants. These interactions can result in the formation of microplastic-associated contaminant aggregates, effectively creating chemical sinks within the marine environment that concentrate pollutants to levels substantially exceeding ambient concentrations [92,93]. The bioavailability of such complexes raises concerns regarding toxicological effects, including carcinogenicity and endocrine disruption [9].

It should be borne in mind that research on micro- and nano-plastics in terms of dietary exposure and health effects is still an emerging topic. Many of the studies are laboratory-based and studies to prove health effects arising from consumption are still the subject of on-going research. It has, however, been established that smaller nano-plastics have greater ability to cross membranes and enter tissues compared to larger micro-plastics and are therefore more likely to result in toxic effects.

The aggregation of plastic debris also leads to the formation of a “plastisphere”, a term describing the microbially active surface communities that develop on marine plastic substrates. This niche ecosystem can facilitate the transport of pathogenic microorganisms, chemical contaminants, harmful algal species, and faecal indicator bacteria, thereby amplifying ecological and public health risks [94,95]. Consequently, the combined presence of chemical and microbial contaminants on plastic particles may enhance the risk of human exposure through the consumption of contaminated fish, crustaceans, and shellfish [96]. Conversely, if these particles are too large to be bio-available, they may actually act to some extent to reduce exposure [97].

## 13. Food Chemical Risk Assessment

While biological hazards often pose a greater short-term risk to consumer health, chemical hazards are generally regarded as the predominant food safety concern [98]. Chemical hazards can be broadly classified into two major categories: (i) residues of agricultural chemicals applied during food production, such as pesticides and veterinary pharmaceuticals; (ii) chemical contaminants. The latter may originate from natural biological processes (e.g., mycotoxins), industrial or anthropogenic activities (e.g., polychlorinated biphenyls [PCBs], dioxins, flame retardants, per- and polyfluoroalkyl substances [PFAS]), or from the geochemical characteristics of the production environment (e.g., lead, arsenic) [99,100].

The principle that the toxicity of a substance depends solely on its dose (“*sola dosis facit venenum*” only the dose makes the poison) was first articulated by Paracelsus (1493–1541). Building upon this foundation, contemporary food safety authorities worldwide have established standardized, science-based risk assessment frameworks to evaluate the potential hazards associated with chemical and biological agents in food [98,100].

Risk assessment encompasses several key stages: hazard identification, hazard characterisation, exposure assessment, and risk characterisation. Hazard identification and characterisation involve determining the nature and toxicological properties of the agent, whereas exposure assessment estimates the extent and frequency of human exposure. The integration of these components through risk characterisation provides a quantitative or qualitative estimate of the overall risk to the population [98,101,102].

Risk managers subsequently interpret the outcomes of scientific risk assessments in the context of economic, social, and political considerations to inform risk management decisions and implement appropriate mitigation measures. Importantly, such management decisions should also account for the nutritional benefits of food consumption; for instance, fish represents a significant source of essential nutrients, and thus, the potential health risks associated with its consumption should be weighed against its demonstrated dietary benefits [100].

The process of food chemical risk assessment is directly relevant for contaminants in fish and seafood and is applied by many regulatory agencies in the same way that they apply it to other food types (e.g., JECFA, EFSA). The main considerations that are specific for these food types are the type of contaminant that is relevant (e.g., POPs, arsenic, mercury) and the vast number of different species consumed and their differences in biology in terms of feeding habits, propensity for bio-accumulation, environmental habitat, etc.

### Regulatory Landscape

The regulatory landscape for contaminants in fish and seafood is shaped by international bodies like JECFA and Codex, which provide scientific advice and set global standards. The EU, US and other regions have robust regulatory frameworks, with EFSA and FDA playing key roles in risk assessment and enforcement. The WTO SPS Agreement promotes harmonization of standards, and national regulations further ensure consumer protection. Together, these systems aim to minimize risks and ensure the safety of fish and seafood worldwide.

## 14. Foods Produced from Aquatic Environments

### 14.1. Fish, Shellfish, and Other Aquatic Animal Species

A fundamental distinction between animal foods derived from terrestrial and aquatic environments lies in the diversity of consumable species. Whereas terrestrial food production is dominated by a limited number of livestock species, aquatic systems provide a much broader range of edible organisms, including numerous fish and shellfish species [34]. Although aquaculture increasingly contributes to the global supply of aquatic foods, the majority of production is concentrated among a relatively small number of farmed species [103].

Food safety concerns associated with fish are often species-specific. Oily or fatty fish tend to accumulate higher concentrations of lipophilic contaminants such as persistent organic pollutants (POPs), including dioxins, polychlorinated biphenyls (PCBs), brominated flame retardants, and legacy pesticides such as DDT [103]. Toxic metals and metalloids, notably mercury and arsenic, bioaccumulate through trophic transfer and are therefore detected at elevated concentrations in predatory fish such as shark, swordfish, and marlin. Both pelagic (surface-feeding) and benthic (bottom-feeding) fish can accumulate contaminants through dietary intake and direct uptake from the surrounding water. However, contamination levels are influenced by local environmental conditions, with benthic species particularly affected by pollutants present in sediments [104,105].

Shellfish differ markedly from finfish in their physiology and feeding behavior. Many species, including oysters, clams, and mussels, are filter feeders that play an ecological role in nitrogen removal by incorporating it into their tissue and shells during growth [106]. However, filter-feeding shellfish are prone to accumulation of polycyclic aromatic hydrocarbons (PAHs), a diverse group of combustion-derived compounds of which several are known to be genotoxic and carcinogenic [103]. While most vertebrate species can metabolize PAHs, shellfish generally lack this capability, resulting in bioaccumulation. PAHs are also associated with thermally processed foods, such as smoked or charred products, e.g., barbecued foods [107].

Marine mammals including cetaceans (e.g., porpoises, dolphins, whales), pinnipeds (e.g., seals, sea lions), sea otters, and sirenians (e.g., manatees, dugongs) occupy high trophic levels and consequently accumulate substantial body burdens of POPs and other bioaccumulative contaminants. These substances can be transferred maternally via placental or lactational pathways [108]. Although such species are primarily studied as indicators of marine pollution, they remain a dietary component in certain human populations. Similarly, reptiles such as sea turtles serve as sentinel species for marine contamination, and both their tissues and eggs are consumed in some regions. Eggs of marine birds, such as gulls, may also reflect contaminant burdens corresponding to their fish-rich diet.

### 14.2. Plant-Derived Aquatic Foods: Seaweeds and Algae

Seaweeds have long been integral to diets in many Asian countries and have gained increasing popularity in Europe over the past decade or so. This trend is driven by consumer shifts toward plant-based, sustainable diets and the perception of seaweed as a health-promoting food [109]. Projections suggest that by 2050, up to 0.1% of oceanic area could be dedicated to seaweed cultivation, yielding approximately fifteen times current global production levels [108].

At present, there are no specific regulatory frameworks governing the safety and quality of seaweed outside Asia. Seaweeds are valued for their nutritional profile, which includes high levels of vitamin B_12_, dietary fibre, omega-3 fatty acids, polyphenols, sulphated polysaccharides, and various pigments linked to potential health benefits. However, seaweeds also have a strong capacity to bioaccumulate minerals and trace elements from surrounding waters. While many of these elements are beneficial, others such as lead, mercury, and arsenic are toxic in certain chemical forms [103]. Iodine, though essential for thyroid hormone synthesis, can lead to thyroid dysfunction when consumed at excessive levels. The European Food Safety Authority (EFSA) recommends an upper tolerable intake of 600 µg iodine per day for adults; thus, consumption of small quantities of iodine-rich seaweed may exceed this threshold [105].

Heavy metals such as mercury, lead, cadmium, and arsenic have no known physiological benefit and are toxic even at low concentrations. Lead is classified as a possible human carcinogen with neurotoxic effects, and inorganic arsenic is designated as a Group 1 carcinogen [110]. Bioaccumulation of these elements varies by seaweed species, environmental conditions (e.g., geographic location, water pollution status, season), and local ecosystem characteristics [108]. Certain species, such as Hijiki seaweed, can contain arsenic at levels high enough to prompt consumption advisories by regulatory authorities [110].

In the example of arsenic in sea-weeds, the arsenic was found to be associated with the water in which it was cooked, and exposure could therefore be minimised if any water used for cooking is discarded. Other contaminants such as POPs are lipophilic and more usually associated with oily fractions, but again if any oils or fats used for cooking are discarded, then exposure to these contaminants can be minimised.

In 2018, the European Commission recommended that Member States undertake monitoring of elemental contaminants in seaweed, with the potential introduction of regulatory limits pending risk assessments conducted by EFSA [105].

## 15. Adulteration in Fish and Aquaculture

Adulteration can occur at various stages of the fish and aquaculture supply chain—from harvesting and processing to packaging and distribution. Common forms of adulteration in this sector include the addition of non-permitted chemicals (undeclared preservatives and colourants), water injection to increase weight, or substitution with lower-value species.

## 16. Environmental Considerations

### Environmental Risk Assessment

The habitat and environment where aquatic food is produced is a critical indicator for the potential contamination of those foods produced. Environmental risk assessment (ERA) is therefore highly relevant for food safety for these foods. It is a systematic process used to evaluate the condition of ecosystems and the broader environment. It plays a crucial role in determining the safety of food products derived from these systems, particularly fish and seafood. Unlike conventional human health risk assessments, which focus on quantifying exposure to hazardous compounds in food by combining hazard characterization with exposure estimation, environmental risk assessments adopt a more holistic perspective that considers the interactions and dynamics within the entire ecosystem.

An ERA integrates a wide range of bioaccumulation markers and biomarkers to assess both exposure to and the biological effects of environmental contaminants [111]. Fish bioaccumulation markers, in particular, are useful for investigating the aquatic behaviour and fate of contaminants. These markers act as bioconcentrators, enabling the detection of substances present at low concentrations in water and allowing assessment of contaminant exposure in aquatic organisms. Such assessments are typically designed to evaluate ecological risks rather than direct risks to human consumers of fish or seafood.

To predict the environmental fate of xenobiotic compounds, models often incorporate parameters such as partitioning behaviour, toxicokinetics, metabolism, biota–sediment accumulation factors (BSAFs), organ-specific bioaccumulation, and bound residues. However, even advanced predictive models have limitations in accurately estimating bioaccumulation in fish; therefore, model outputs should be supported by empirical analytical data [112].

Key indicators of environmental health in fish include body burdens of persistent organic pollutants (POPs), such as dioxins, polychlorinated biphenyls (PCBs), brominated flame retardants (BFRs), and dichlorodiphenyltrichloroethane (DDT). In contrast, more readily biodegradable compounds such as polycyclic aromatic hydrocarbons (PAHs) and chlorinated phenols tend not to accumulate in fish tissues at levels that accurately reflect exposure, thereby limiting their usefulness as bioaccumulation markers [111,112]. Practical regulatory approaches and benchmarking methods are increasingly used to interpret bioaccumulation data from fish studies for decision-making in chemical assessment [113].

## 17. Water-Table Contamination

Although this review primarily addresses contaminants in fish and seafood, it is important to recognize that the safety of all food commodities is fundamentally dependent on water quality. A notable example of food contamination arising from water-related issues is the occurrence of arsenic in agricultural produce, particularly in regions where arsenic is naturally present in the geological strata [114].

Arsenic contamination of groundwater is a naturally occurring phenomenon in several parts of the world, with South Asia being one of the most severely affected regions [115]. The use of arsenic-contaminated water for drinking, food preparation, and irrigation poses a major public health concern due to the toxic nature of arsenic, particularly in its inorganic forms, as discussed previously in relation to metal(loid) contamination in fish [116].

Historically, prior to the 1970s, Bangladesh experienced one of the highest global infant mortality rates, largely attributable to microbial contamination of surface water sources. Inadequate sanitation infrastructure, inefficient water purification systems, and the impacts of seasonal monsoons and flooding exacerbated this problem. In response, UNICEF and the World Bank promoted the installation of tube wells to access deeper, ostensibly safer groundwater sources. This initiative resulted in the construction of over eight million wells and led to a 50% reduction in infant mortality and diarrheal diseases [117].

However, subsequent investigations revealed that many of these wells were drawing water from arsenic-rich geological formations within the Ganges Delta. Approximately 20% of the wells exceeded the national drinking-water standard for arsenic concentration. A global assessment conducted in 2007 estimated that over 137 million individuals in more than 70 countries were exposed to arsenic-contaminated drinking water. Although mitigation efforts have since reduced the number of people exposed above the Bangladesh standard, groundwater contamination remains a widespread issue. The contamination of irrigation water consequently extends to agricultural produce, particularly rice—a major export commodity from the region—thereby creating a transboundary food safety concern [115,116].

This example is included to illustrate that water quality and pollution in water can have an impact that is much wider than fish, seafood and other foods directly produced in marine, freshwater and aquaculture environments and can in fact impact all food types since water is essential for all animal and plant foods produced worldwide.

## 18. Risk Substitution

All water supply systems inherently carry health-related risks, and interventions aimed at mitigating one hazard may inadvertently introduce another, sometimes of greater severity [118]. The situation in Bangladesh exemplifies such “risk substitution”: measures implemented to reduce microbial contamination by promoting shallow groundwater use unintentionally increased population exposure to arsenic in tubewell water [119,120].

Effective emergency responses to arsenic contamination must therefore be designed within a comprehensive risk management framework, such as the Water Safety Plan approach, which integrates hazard identification, risk assessment, and mitigation strategies across all stages of water supply [121,122]. A consistent methodology for evaluating and prioritising risks is essential to ensure that interventions do not simply replace one hazard with another [118].

Potential substitute hazards include microbial pathogens, cyanobacterial toxins present in surface waters, and chemical pollutants from anthropogenic sources [122]. More broadly, the principle of risk substitution underscores the necessity for holistic water resource management that considers the full spectrum of potential hazards when developing and implementing contamination control measures [118,121].

## 19. Research Gaps and Future Direction

### 19.1. Risk-Benefit Analysis and Personalised Medicine

Elevated concentrations of chemicals of concern, such as polychlorinated biphenyls (PCBs) and dioxins, are frequently detected in species that are also rich in essential nutrients, notably vitamin D and omega-3 fatty acids. While conventional risk assessment methodologies for evaluating hazards to human health are well established, and environmental risk assessment is comparatively developed, comprehensive assessments that balance risks and benefits remain uncommon. Nevertheless, such analyses are particularly critical when considering the consumption of fish and seafood. Persistent organic pollutants (POPs) tend to accumulate in oily fish, which simultaneously serve as significant sources of vitamin D and omega-3 fatty acids. Recommendations to reduce oily fish intake, especially in regions with limited sunlight, may inadvertently exacerbate vitamin D deficiency, particularly during winter months. Although the avoidance of oily fish may be suggested to mitigate exposure to carcinogenic contaminants, this strategy could diminish the cardiovascular protective effects conferred by omega-3 fatty acids. Such guidance may be appropriate for individuals with a heightened cancer risk and a lifestyle or genetic profile indicating low susceptibility to heart disease; conversely, it may not be optimal for those with a lower cancer risk but greater predisposition to cardiovascular conditions. Public health recommendations intended to safeguard the general population may prove beneficial for the majority, yet they carry the potential to adversely affect certain individuals. Thus, risk-benefit analysis and the application of personalised medicine represent vital areas for future research to ensure that dietary advice is both effective and appropriately targeted.

### 19.2. Risk Assessment of Mixtures

Chemical contaminants are seldom present as isolated entities within environmental matrices. Regulatory risk assessments and the establishment of control thresholds predominantly focus on individual contaminants or discrete contaminant classes, often neglecting the complexities associated with exposure to mixtures. Although advancements have been made in evaluating the risk of compounds with analogous toxicological mechanisms such as dioxins and dioxin-like polychlorinated biphenyls (PCBs), which exert their effects via the aryl hydrocarbon (AhH) receptor, progress remains limited. Efforts to conduct risk assessments for endocrine-disrupting chemicals have also been initiated. Nevertheless, the co-occurrence of contaminant classes with markedly divergent modes of action, such as arsenic, mercury, dioxins, and polyfluorinated alkyl substances, presents significant methodological challenges. These substances are frequently detected concomitantly in aquatic organisms, including fish. Consequently, the development of robust frameworks for the assessment of mixed contaminant exposures should be prioritised to enhance the protection of both human and environmental health.

### 19.3. Microplastics and Nanoplastics

The issue of microplastics and nanoplastics has been previously addressed within this review. Research in this domain remains nascent, with substantial challenges persisting. Critical methodological gaps include the identification and characterisation of plastic types comprising environmental mixtures, the evaluation of toxicity, determination of overall absorption and bioavailability, and elucidation of the influence of adsorbed chemical pollutants on particle surfaces. Further investigation is required to address these topics and to advance understanding of the risks posed by micro- and nanoplastic contaminants to aquatic species and, by extension, to human health.

### 19.4. Algae

Algae are increasingly recognised as a valuable resource for food production, offering considerable potential for exploitation as foodstuffs and functional ingredients. Comprehensive scientific investigation is required to elucidate the full spectrum of applications for these diverse plant materials in the development of novel food products, whilst concurrently ensuring the safety and quality of the resulting foods [10].

### 19.5. Climate Change and Impact of Flooding

Elevated sea surface temperatures exert significant influence on the biogeochemical cycling of mercury, particularly affecting the transformation of inorganic mercury into the more toxic methylmercury species [20,123,124]. Methylation of mercury in aquatic environments is facilitated by both microbial and chemical processes, with increased water temperatures enhancing these mechanisms. Empirical estimates indicate that methylmercury uptake by fish and marine mammals may rise by approximately 3–5% for each 1 °C increment in water temperature, thereby amplifying the risk of human exposure through dietary intake of fish [125].

A warmer climate may result in increased atmospheric transport of POPs associated with dust particles towards polar regions away from warmer regions. Reduced ice caps may result in increased release from a greater area of exposed ocean waters, and more pollutants may be formed associated with smoke from an increased number of forest fires.

There may be more potential for pollutants to become available from sources that were previously buried, from sources such as beneath melting glaciers and permafrost [126].

Top predators may need to change their diet in response to changes in environment such as loss of sea ice habitats and changes in associated diets. Some species may migrate northwards in response to climate changes resulting in changes to the food web [126].

Flooding events serve as vectors for the dispersion of environmental contaminants, including dioxins, heavy metals, and hydrocarbons, from polluted to previously uncontaminated regions. Notably, the 2002 flooding of the Elbe and Mulde rivers in central Europe resulted in the redistribution of dioxin-laden soil onto adjacent floodplains [17]. Similar occurrences have been documented in river systems traversing urban and industrial landscapes in the United Kingdom. Subsequent analyses revealed increased dioxin concentrations in milk produced by cows grazing on these contaminated floodplains in comparison to those fed in barns or on control pastures, underscoring the potential threat to food safety posed by grazing livestock on polluted land. Effects relating specifically to brominated flame retardants in view of climate change were recently considered by [127].

The aftermath of Hurricane Katrina further exemplified the risk of widespread chemical contamination arising from floodwaters, which mobilised pollutants from compromised sewage treatment facilities, oil refineries, chemical plants, storage depots, and inadequately managed hazardous waste sites. Post-hurricane assessments by the United States Environmental Protection Agency (USEPA) in 2005 detected elevated concentrations of arsenic, lead, and polycyclic aromatic hydrocarbons (PAHs) in a subset of the 1800 sediment and soil samples analysed. The highest arsenic levels were attributed to historical herbicide application, while localised PAH contamination near landfill sites suggested spillage, and elevated lead concentrations reflected the mobilisation of legacy pollutants during flooding events [128].

### 19.6. Cross Boundary Management/Considerations

Numerous principal river systems serve as international boundaries or traverse multiple nations throughout their courses. This facilitates transboundary transport of pollutants, particularly between countries governed by disparate regulatory frameworks and environmental management practices, thereby exacerbating potential diplomatic tensions [129]. The construction of dams for water storage or the diversion of river water for irrigation purposes can precipitate supply deficiencies in downstream regions, often leading to disputes over equitable water allocation [130]. Escalating population growth and increasing demands on freshwater resources have led to projections that water scarcity may emerge as a catalyst for future international disputes and conflicts [131]. Accordingly, the establishment of comprehensive international agreements governing the management of shared water resources is likely to be crucial for mitigating the risk of droughts and preventing conflict [129,130].

## 20. Conclusions

This review has highlighted the complex interplay between the nutritional benefits and the risks associated with consuming foods from aquatic environments, including both wild-caught and farmed products. Aquatic foods are important sources of high-quality protein, essential fatty acids, vitamins, and trace elements, contributing significantly to global food security and public health. However, these benefits are counterbalanced by the presence of a wide array of chemical contaminants—such as pesticides, veterinary medicines, persistent organic pollutants (POPs), heavy metals, radionuclides, microplastics, and natural biotoxins—that can pose significant risks to both human health and ecosystem integrity.

The expansion of aquaculture has helped alleviate pressure on wild fish stocks but has also introduced new challenges related to environmental contamination and food safety. The review underscores the importance of integrated monitoring and regulatory frameworks to manage contaminant levels, particularly as climate change and anthropogenic activities continue to alter the distribution and toxicity of hazardous substances in aquatic systems.

Key recommendations include the need for robust risk–benefit analyses to inform dietary guidelines, and the development of regulatory controls for emerging contaminants such as micro- and nanoplastics. The review also calls for improved international cooperation to address transboundary pollution and water resource management, as well as ongoing research into the risks posed by contaminant mixtures and the potential of algae and other novel aquatic foods.

Ultimately, safeguarding the safety and sustainability of aquatic food systems requires a holistic approach that balances nutritional benefits against chemical and biological hazards, integrates environmental and food safety risk assessments, and adapts to evolving challenges posed by climate change and globalisation. By advancing scientific understanding and policy development in these areas, stakeholders can better protect public health and ensure the long-term viability of aquatic food resources.

## Figures and Tables

**Table 1:** Ways in which veterinary pharmaceuticals are integral to modern aquaculture.

Purpose/Scenario	Role of Veterinary Pharmaceuticals
Enhancing biosecurity and husbandry	Vaccines, disinfectants, and prophylactic agents form the foundation of robust health management, helping to prevent disease introduction and spread.
Treating production-limiting diseases	Therapeutic interventions minimize losses from reduced growth, poor feed conversion, and increased mortality caused by infections and parasites.
Managing epizootic disease outbreaks	Swift, effective use of veterinary medicines is crucial for controlling large-scale outbreaks and averting the collapse of operations or even entire industry sectors [34].
Early cultivation of new species	Used where pathogen–host interactions are not fully understood.
When preventive measures fail	Applied in situations where vaccination or optimal husbandry do not prevent disease.
Managing emerging or re-emerging diseases in changing production environments	Used to address new or returning diseases as production environments evolve.
Responding to environmental and climatic shifts	Used when shifts affect pathogen distribution and virulence.
Ensuring animal welfare	Helps meet evolving standards for the health and treatment of cultured species [34].

**Table 2:** Concerns regarding the overuse or misuse of veterinary medicines in aquaculture.

Concern/Issue	Description
Diagnostic challenges	Effective disease control depends on accurate, timely diagnosis before administering medicines. Without rapid diagnostics, treatments are often empirical or prophylactic, increasing risk of misuse and antimicrobial resistance (AMR).
Human and animal health risks	The emergence and spread of AMR among bacteria in aquatic environments and humans is a serious concern. Residues of banned or unregulated substances in aquaculture products may also pose direct health risks, such as toxic or allergic reactions. Concentrations exceeding established Maximum Residue Limits (MRLs) are hazardous, and residues can disrupt human gut flora and promote resistance in enteric pathogens [35,36].
Environmental and ecological impacts	Veterinary medicines can enter the environment through uneaten medicated feed, effluent discharge, or excreted residues, accumulating in sediments and aquatic ecosystems. Consequences include changes in microbial communities, toxicity to non-target species, and the spread of AMR genes among aquatic bacteria [35].
Legislative and regulatory limitations	Sustainable use of veterinary medicines requires strong legal and regulatory frameworks. National authorities must regulate drug registration, prescription, licensing, and record-keeping, and ensure enforcement through adequate capacity and resources. The absence of such frameworks contributes to misuse and over-use of veterinary medicines in aquaculture [35].

**Table 3:** Key Contaminants in Fish and Seafood.

Contaminant Type	Examples & Sources	Health Risks/Notes
Pesticides	Organophosphates, carbamates, pyrethroids, DDT, lindane, chlordane; agricultural runoff, direct use in aquaculture	Endocrine disruption, neurotoxicity, carcinogenicity, developmental effects; bioaccumulation in food web
Veterinary Medicines	Antimicrobials, antiparasitics, organophosphates, pyrethroids; used in aquaculture and livestock	Antimicrobial resistance, toxic/allergic reactions, residue accumulation, environmental contamination
Persistent Organic Pollutants (POPs)	Dioxins, PCBs, PBDEs, PFAS, BFRs, organochlorine pesticides; industrial/agricultural sources, contaminated feed	Carcinogenicity, immunotoxicity, neurotoxicity, endocrine disruption; bioaccumulation and biomagnification
Heavy Metals & Metalloids	Mercury (methylmercury), cadmium, lead, arsenic; industrial discharge, mining, runoff	Neurological deficits, renal/hepatic damage, reproductive toxicity, carcinogenicity; methylmercury in predatory fish
Radionuclides	Caesium-137, strontium-90, iodine-131; nuclear fallout, accidents, contaminated water	Cancer risk, thyroid dysfunction, bone disorders; bioaccumulation in aquatic organisms
Natural Biotoxins	Saxitoxins, domoic acid, ciguatoxins, tetrodotoxin; produced by algae, present in some fish/shellfish	Paralytic, amnesic, ciguatera fish poisoning; neurological and gastrointestinal symptoms
Mycotoxins	Aflatoxins, ochratoxins, fumonisins; from contaminated aquafeeds (plant-based)	Immunosuppression, carcinogenicity, organ toxicity
Polycyclic Aromatic Hydrocarbons (PAHs)	From combustion, smoked/charred foods, environmental pollution	Genotoxicity, carcinogenicity; bioaccumulation in shellfish
Microplastics & Nanoplastics	Plastic debris, synthetic fibers, tire wear particles; riverine/marine transport, aquaculture	Poorly understood toxicity, potential for adsorbing other pollutants, ingestion by filter feeders and humans
Eutrophication-related Toxins	Harmful algal blooms, cyanobacterial toxins; nutrient runoff from agriculture	Shellfish poisoning, fish kills, ecosystem disruption
Excess Iodine (from seaweed)	Iodine-rich seaweed, especially Hijiki; bioaccumulation from water	Thyroid dysfunction if consumed in excess

## Data Availability

No new data were created or analyzed in this study.
